# IL-33 Induces IL-9 Production in Human CD4^+^ T Cells and Basophils

**DOI:** 10.1371/journal.pone.0021695

**Published:** 2011-07-06

**Authors:** Lars Blom, Britta C. Poulsen, Bettina M. Jensen, Anker Hansen, Lars K. Poulsen

**Affiliations:** 1 Dermato-Allergological Department K, Copenhagen University Hospital Gentofte, Gentofte, Denmark; 2 Department of Veterinary Disease Biology, Faculty of Life Sciences, University of Copenhagen, Copenhagen, Denmark; McGill University, Canada

## Abstract

IL-33, an IL-1 family member and ligand for the IL-1 receptor-related protein ST2, has been associated with induction of Th2 cytokines such as IL-4, IL-5, and IL-13. Here, we report that IL-33 can initiate IL-9 protein secretion *in vitro* in human CD4+ T cells and basophils isolated from peripheral blood. TGF-β has been described as a critical factor for IL-9 induction in Th2 cells; however, we found that TGF-β also induces co-production of IL-9 in purified, naïve (>99%) CD4^+^CD45RA^+^CD45RO^−^CD25^−^ T cells differentiated towards a Th1 profile. Subsequently, it was demonstrated that TGF-β is important, although not an absolute requirement, for IL-9 production in CD4+ T cells. IL-9 production by purified (>95%) human basophils, cultured for 24 h with IL-3 or IL-33, was found, with a strong synergy between the two, likely to be explained by the IL-3 upregulated ST2 expression. Collectively, these data indicate that barrier functioning cells are important for the regulation of IL-9 production by immune cells in inflamed tissue.

## Introduction

A broad spectrum of pathogens activates innate immune responses that direct a specific adaptive immune response. Depending on the cytokine milieu and type of pathogen, naïve CD4+ T cells differentiate into specialized subsets, such as Th1, Th2, Th17, T follicular, or T regulatory (Treg), all important in different stages of the immune response [1]. Each CD4+ T cell subset is characterized by the expression of lineage-specific transcription factors, and produces a repertoire of signature cytokines, such as IFN-γ or IL-4, IL-5 and IL-13 for Th1 and Th2 cells, respectively. A fine tuning of the cytokine profile acquired by the differentiating T cells in the lymph node or other immune cells in the tissue is hypothesized to take place after homing to inflamed tissue, influenced by local alarming cytokines, such as IL-25, IL-33, and thymic stromal lymphopoietin [2]. These alarming cytokines may be released not only by immune cells, but also by epithelial cells upon encountering allergens, helminth-derived products, viruses, or other irritants causing stress [2]. IL-33 is mainly secreted by lung barrier functional cells, such as fibroblasts, epithelial cells, and endothelial cells [3]. IL-33 an IL-1 family member together with IL-1β and IL-18 mediates its biological effects via IL-1 receptor-like 1 (ST2) and has been reported to trigger production of the main allergy signature cytokines IL-4, IL-5, and IL-13 by Th2 cells, basophils, and mast cells [4]–[7]. It has been hypothesized that IL-33 is released after tissue damage by, for instance, allergens, resulting in necrotic cell death [8]; thus, linking the release of IL-33 to an activation of the surrounding cells such as T cells or basophils expressing ST2 [9].

In addition to the described alarming factors, the pleiotropic cytokine TGF-β has been associated with differentiation of various CD4+ T cell subsets, including Treg, Th9, and Th17; and TGF-β has been described to be important in airway remodelling and tissue repair [1], [10]. In asthmatics, TGF-β mRNA expression was increased in bronchial airways and it has been shown that prolonged allergen challenge induces production of active TGF-β in lung tissue [11]. TGF-β in relation to differentiation of CD4+ T cells into a Th9 subset was first described by Schmitt et al., who showed that TGF-β and IL-2 induce IL-9 in a subpopulation of Th2 cells, later classified as Th9 cells [12]. Various cell types involved in allergy and asthma are known to express the IL-9 receptor, however the physiological role of IL-9 is currently not well understood [13]. Expression of IL-9 has been found to be significantly increased in atopic patients compared to non-atopic subjects, and, in addition, it has been suggested to be important in chronic allergic inflammation [14], [15].

Until recently, there has not been described a specific master transcription factor for the Th9 subpopulation; however, in 2010 Chang *et al.* reported that PU.1 is expressed specifically in the IL-4^low^IL-9^high^ population of Th2 cells, separating this subpopulation into a distinct lineage [14]. The *in vitro* regulation of IL-9 in CD4+ T cell cultures has been found to be influenced by multiple cytokines, including IL-1β, IL-2, IL-4, IL-6, IL-10, IL-21, IL-23, IFN-α, IFN-β, and TGF-β [5], [12], [16], [17].

The number of cytokines regulating IL-9 production in CD4+ T cells is increasing in parallel with the number of CD4+ T subsets that have been discovered to produce IL-9, including Th2, Th9, and Th17 [5], [16], [17]. Here we report that IL-33 independent of TGF-β upregulates IL-9 secretion in human *in vitro* differentiated CD4+ cells. This may reflect a more general inflammatory pathway, since our results also showed that IL-33 in synergy with IL-3 induces secretion of IL-9 in *in vitro* cultured human basophils. This might imply that IL-33 has an important role in the regulation of IL-9 in the tissue immune cells.

## Materials and Methods

### Isolation of naïve CD4+ T cells and basophils

Cells used in this study originate from buffy coat fractions of blood portions from anonymized donors, whose identity has been anonymized for the researcher by the Blood Bank of the National University Hospital Copenhagen (Copenhagen, DK).

PBMC were purified using lymphoprep density centrifugation and hemolysis of remaining erythrocytes was performed on ice. The cells were washed twice with PBS^+^ buffer (PBS without Ca^2+^ and Mg^2+^) (Invitrogen) and supplemented with 0.5% (vol./vol.) EDTA (Bie & Berntsen, Rødovre, DK) and 0.5% (vol./vol.) human serum albumin (ZLB Behring GmbH, Marburg, DE).

Using the Naive CD4+ T Cell Isolation Kit II (Miltenyi, Gladback, DE), according to the manufacturer's instructions, naïve CD4^+^CD45RA^+^CD45RO^−^CD25^−^cells were magnetically isolated. In short, PBMC were incubated with a cocktail of biotinylated antibodies (Miltenyi), followed by labeling with magnetic anti-biotin-coated microbeads for magnetic depletion. The cells were depleted twice using new LS columns for each round of depletion, and subsequently stained and depleted with the same cocktail of biotinylated antibodies and anti-biotinylated microbeads to ensure a purity of >99% CD4^+^CD45RA^+^CD45RO^−^CD25^−^ cells.

Basophils were purified from the PBMC fraction, as described above without hemolysis, using the Basophil Isolation Kit II (Miltenyi) according to the manufacturer's protocol. In brief, Fc receptor blocking reagent was applied along with biotin-conjugated antibodies. Following incubation, anti-Biotin magnetic beads were added, and after a second incubation, the cells were washed and applied to a LS column placed in a magnet. The basophils were eluted by washing the column with cold PBS. Throughout the purification process the basophils were kept at temperatures <8°C. The purity of the isolated basophils used for FACS analysis of ST2 expression was 89.7% ±8.8, whilst the purity of isolated basophils used for multiplex analysis was 95.5% ±0.8.

### Cultures

Naïve CD4+ T cells were cultured at 37°C in 6- or 48-well flat-bottomed plates from Nunc (Roskilde, DK) in RPMI 1640 from Sigma (Saint Louis, MO, USA) supplemented with 100 U/ml penicillin and 100 µg/ml streptomycin, 1 mM L-glutamine from Invitrogen (Carlsbad, CA, USA), 50 µM β-mercaptoethanol (Sigma), and 5% (vol./vol.) human AB serum from Righospitalet (Copenhagen, DK). The naïve CD4+ T cells, were cultured at 1*10∧5 cells per ml in 500 µl media and supplemented with irradiated murine CD32-transfected fibroblasts (162GC-7) by GAMMACELLE 2000 RH from AEK Risø (Risø, DK) at a ratio of one fibroblast per five T cells, in the presence or absence of the indicated human cytokines added day 0 and/or 5∶10 ng/ml IL-4, IL-12, and IL-33 from eBioscience (San Diego, CA, USA), 10 ng/ml IL-2 Proleukin from Novartis (Auckland, AUS), and 10 ng/ml IL-1β, IL-18, and TGF-β from Humanzyme (Chicago, IL, USA). Antibodies used: 5 µg/ml anti-IL-12, and 5 µg/ml anti-PAN specific TGF-β from R&D systems (Minneapolis, MN, USA), 10 µg/ml anti-IL-4 (MP4-25D2), and 5 µg/ml anti-IFN-γ (MD-1, eBioscience), 30 ng/ml anti-CD3 (UCHT1), and 30 ng/ml anti-CD28 (CD28.2) from BD Biosciences (Franklin Lakes, NJ, USA). The cultures were restimulated day 5. At day 8, the cultures were split into two and medium was added 1∶1 to obtain cultures for either quantitative RT-PCR (qRT-PCR) or FACS and multiplex analysis.

Cultures for FACS and multiplex analysis were re-stimulated for 6 hours with 25 ng/ml 13-phorbol 12-myristate acetate (PMA), and 1 µg/ml ionomycin, and for the last 4 hours of incubation also 10 µg/ml Brefeldin A (Bref A) (Sigma).

Cell pellets for qRT-PCR analysis of mRNA were snap-frozen in liquid nitrogen.

Human basophils were cultured at 37°C for 24 hours in RPMI 1640 from (Sigma) supplemented with 100 U/ml penicillin, 100 µg/ml streptomycin, and 1 mM L-glutamine (Invitrogen), and 5% (vol./vol.) human AB serum (Righospitalet). Purified basophils were resuspended in preheated media to a concentration of 1*10^6^ cells/ml, and cultured in 1.5 ml eppendorf tubes with 0.3 ml cell suspension in each tube. Concentration of IL-3 was 10 ng/ml (R&D), and IL-33 50 ng/ml (eBioscience). After 24 h of culture, 200 µl of the cell suspension was used for FACS analysis and the remaining cells were centrifugated at 1000 *g* for 5 min, the supernatant was removed and saved for multiplex analysis, and the pellet was snap-frozen in liquid nitrogen for qRT-PCR analysis. All samples were stored at −80°C.

### Multiplex supernatant analysis

Supernatants from the T cell cultures were assessed for the cytokine secretion of; IL-4, IL-5, IL-9, IL-13, TGF-β1, and IFN-γ, and the supernatant from basophils for IL-4, IL-5, IL-9, and IL-13 secretion by multiplex magnetic bead assay (BioRad, Berkeley, CA, USA), according to the manufacturer's protocol, with the exception of no acid activation in the TGF-β analysis. The samples were analyzed on a Bio-Plex 200 System using Bio-Plex Manager 5.0 (BioRad).

### Flow cytometry

At day 10, the T cells were restimulated and subsequently harvested and washed in PBS^+^ and reconstituted in PBS without Ca^2+^ and Mg^2+^ at a concentration of 1*10∧6 cells per ml. Staining with a LIVE/DEAD fixable red dead cell stain kit (Invitrogen) was performed according to the manufacturer's protocol. The cells were reconstituted in 100 µl PBS^+^ and surface stained with anti-CD4 conjugated with either PE-cy5 (PC5) or PE-cy7 (PC7), both (OKT4) from Biolegend (San Diego, CA, USA) for 15 min in the dark on a rocking table, at room temperature. The cells were washed in PBS^+^, fixed, and permeabilized with IntraPrep from Beckman Coulter (Fullerton, CA, USA) according to the manufacturer's protocol, and subsequently stained according to the surface procedure, using different mix of fluorescently labeled antibodies against: anti-IL-9-PE (MH9A4), anti-IL-13- peridinin chlorophyll protein complex (PerCP) (JES10-5A2), anti-IFN-γ-FITC (4S.B3), and anti-T-bet-FITC (4B10, Biolegend), anti-IL-4-PE-cy7 (8D4-4), anti-GATA3-PE (TWAJ, eBioscience), anti-IL-5-FITC (9906, R&D), and also anti-PU.1-Alexa 488 (9G7) from Cell Signaling (Danvers, MA, USA). The cells were first gated on the LIVE region, second the CD4+ region followed by analysis of intracellular cytokines or transcription factors. A cutoff of 2% of the isotype control was used for the surface and the transcription factors, whereas the cytokines were primarily separated by gating on populations.

To assess the purity of freshly isolated basophils, the cells were resuspended to a concentration of 5*10^5^ cells/ml in 100 µl PBS^+^, and surface stained with PC7-conjugated CD3, CD14, and CD19 (Biolegend) and CD56 (Beckman Coulter); and FITC-conjugated FcεRIα (Biolegend) for 30 min, at 4°C, in the dark. Basophils were gated as FcεRIα^+^CD3^−^CD14^−^CD19^−^CD56^−^. In addition, both freshly isolated and cultured basophils were stained with FITC-conjugated ST2 (MBL) and mouse IgG1 (Beckman Coulter), and expression of ST2 for all samples was compared to their respective isotype control. Expression of ST2 at 24 h was analysed by comparing the expression level of ST2 in the negative control culture with that of the stimulated control cultures.

All labeled cells were analyzed by FACS using a Beckman Coulter FC500 MPL flow cytometer with CXP software (Beckman Coulter). Amine reactive compensation beads or compensation beads (Invitrogen) were used according to the manufacturer's protocol for each antibody for compensation of the FACS data prior to data analysis using FlowJo from Treestar (Ashland, OR, USA).

### RNA purification

RNA was purified using RNeasy mini kit from Qiagen (Hilden, DE) according to the manufacturer's protocol. RNA concentrations were measured using an RNA determination kit on a Qubit Flurometer (Invitrogen).

### cDNA synthesis

0.5 or 0.1 µg total RNA from CD4+ cell or basophil cultures, respectively, was mixed with 1 µl of 0.5 µg/ml oligo (dT) (Invitrogen) in a total volume of 12 µl and incubated for 10 minutes at 70°C to denature the RNA. The RNA was transcribed to cDNA using Superscript II reverse transcriptase (Invitrogen) at 42°C for 50 minutes followed by heat inactivation of enzymes at 70°C for 10 min. The cDNA was stored at −20°C.

### qRT-PCR

Relative mRNA expression levels were determined by qRT-PCR using the ABI PRISM 7700 sequence detection system from Applied Biosystems (Carlsbad, CA, USA). For each triplicate qRT-PCR analysis, 1.5 µl cDNA was mixed with 12 µl water, 15 µl Universal PCR master mix and 1.5 µl pre-developed Taqman assay (Applied Biosystems) specific for either IL-4 (#Hs99999030_m1), IL-5 (#4327039T), IL-9 (#Hs00174125_m1), GATA-3 (#Hs00231122_m1), ST2 (#Hs00249389_m1), PU.1 (#Hs00231368_m1), T-bet (#Hs00203436_m1), TGF-β1 (Hs00171257_m1), β-actin (#401846), or huPO (#4326314E). The mixture was vortexed slightly and run in triplicates in a Taqman ABI PRISM Sequence 7700 with Sequence Detector v1.7a software (Applied Biosystems).

Data were analyzed by setting baseline interval and threshold. The default setting was 3 to 15 cycles for baseline and 10 times standard deviation for threshold, and analyzed using the ΔΔ cycle threshold (CT) method [18].

A calibrator stock was made by purifying PBMC from four buffy coats and stimulating different PBMC cultures overnight with either 5 µg/ml Staphylococcal Enterotoxin B, 5 ng/ml PMA, 1 µM ionomycin all (Sigma), or 1 µg/ml plate-bound anti-CD3 from DAKO (Glostrup, DK). The PBMC were snap-frozen in liquid nitrogen and RNA was harvested and used in cDNA synthesis reactions. The cDNA from the three different stimulations were combined and stored at -80°C.

### Statistical analysis

Bar graphs are represented as mean +/− SEM. One-way matched Bonferroni ANOVA was used for statistical analysis; data from qRT-PCR and supernatant were log transformed prior to analysis. Statistics were performed using Prism 4.0 from GraphPad (San Diego, CA, USA).

## Results

### TGF-β induces IL-9 production in Th1 and Th2 cells

It has been reported that TGF-β inhibits classic Th1 and Th2 skewing, and furthermore induces a Th9 phenotype when added to Th2 cultures. To confirm this and to additionally investigate whether TGF-β can induce IL-9 in differentiated Th1 cultures we developed an *in vitro* system with delayed addition of TGF-β, to established Th1 and Th2 cultures.

The Th1 and Th2 cultures were established at day 5 (Figs. S1B, C, E and F; S2, B and C), with little secretion and expression of IL-9 in the Th2 cultures (Figs. S1, A and D; S2A). In addition no *PU.1* gene expression (Fig. S2D) could be detected in any of the cultures, nor the active form of TGF-β1 in the supernatant (Fig. S1G) even though all cultures expressed the *TGF-β1* gene (Fig. S2D).

**Figure 1:**
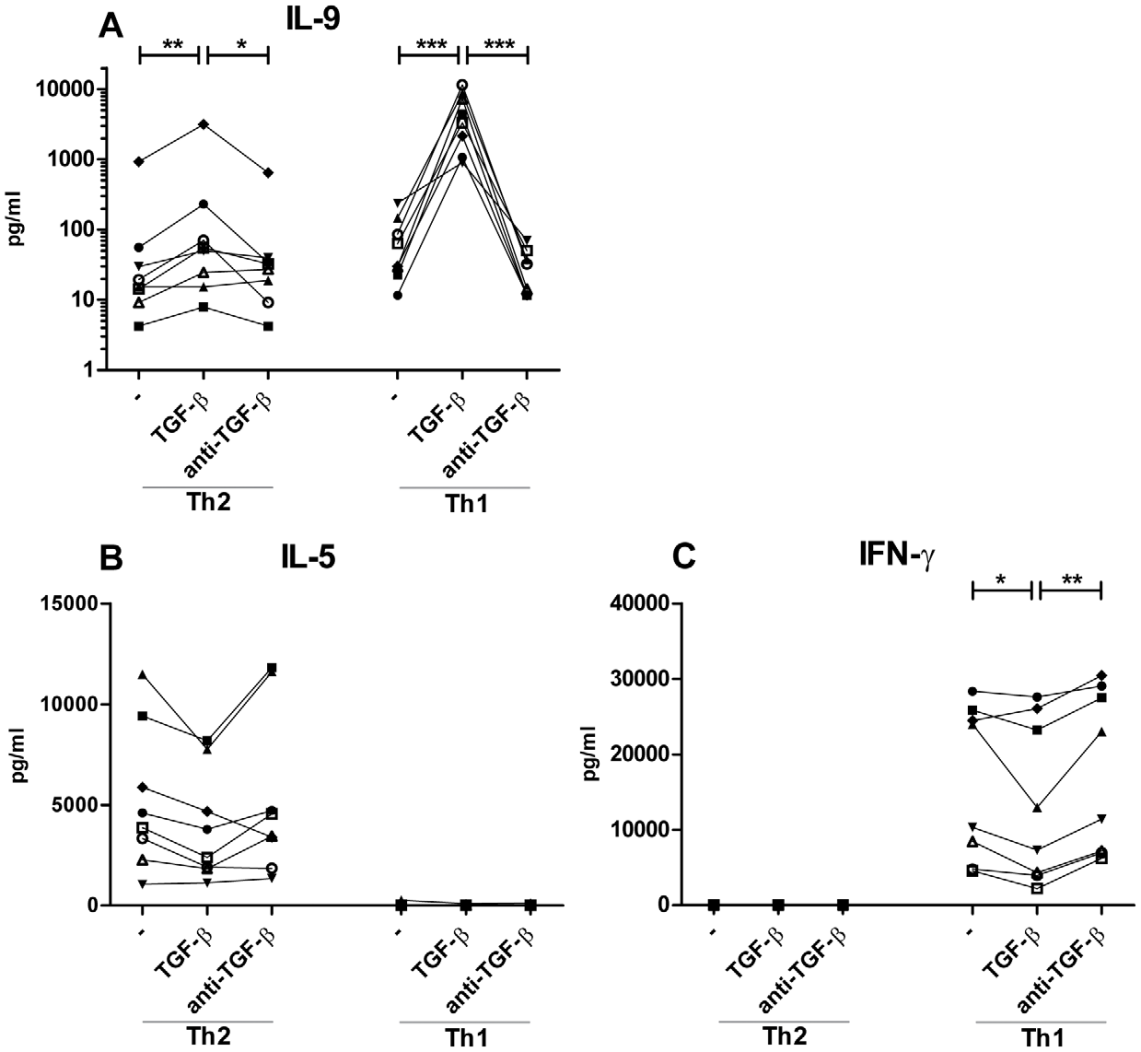
TGF-β induces IL-9 secretion in Th1 and Th2 cells. Naïve CD4+ T cells were activated with fibroblast-bound anti-CD3/CD28 under classical Th1 and Th2 conditions, with IL-12 and anti-IL-4 or IL-4, anti-IFN-γ, and anti-IL-12 respectively for 5 days, restimulated at day 5 and split in cultures with and without TGF-β or anti-TGF-β for 5 more days of stimulation. (A, B and C) Supernatant concentrations of IL-9, IL-5 and IFN-γ at day 10 after restimulation with PMA and ionomycin for 6 h in the presence of Bref A for the last 4 h. Each donor is represented by a specific symbol and connected with a line. Data are from four independent experiments, each with two donors. * *p*<0.05, **p<0.01, ***p<0.001.

**Figure 2:**
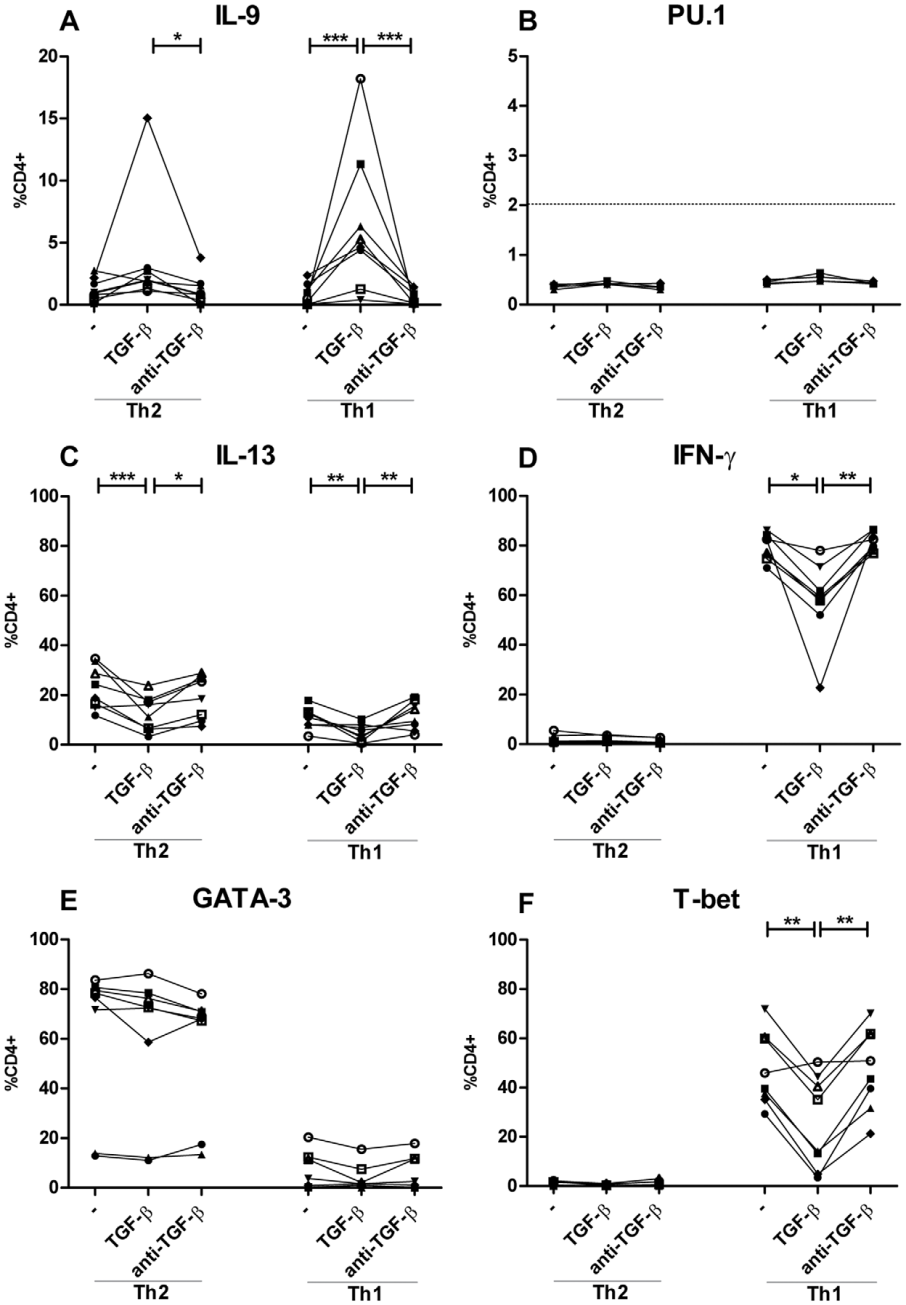
TGF-β induces IL-9 expression in Th1 and Th2 cells. Naïve CD4+ T cells were activated with fibroblast-bound anti-CD3/CD28 under classical Th1 and Th2 conditions, with IL-12 and anti-IL-4 or IL-4, anti-IFN-γ, and anti-IL-12 respectively for 5 days, restimulated at day 5 and split in cultures with and without TGF-β or anti-TGF-β for 5 more days of stimulation. (A, B, C, D, E and F) Percentage positive LIVE^+^CD4+ cells for, respectively IL-9, PU.1, IL-13, IFN-γ, GATA-3, and T-bet at day 10 after restimulation with PMA and ionomycin for 6 h in the presence of Bref A for the last 4 h. Each donor is represented by a symbol and connected with a line. Data are from four independent experiments, each with two donors.* *p*<0.05, **p<0.01, ***p<0.001.

**Figure 3:**
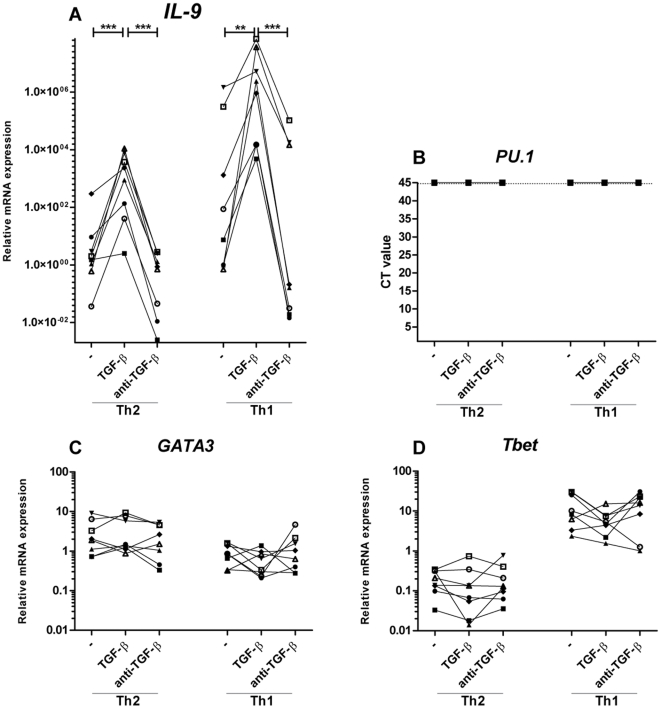
TGF-β induces IL-9 mRNA expression in Th1 and Th2 cells. Naïve CD4+ T cells were activated with fibroblast-bound anti-CD3/CD28 under classical Th1 and Th2 conditions, with IL-12 and anti-IL-4 or IL-4, anti-IFN-γ, and anti-IL-12 respectively for 5 days, restimulated at day 5 and split in cultures with and without TGF-β or anti-TGF-β for 5 more days of stimulation. (A, C and D) qRT-PCR gene expression analysis of the relative gene expression of *IL-9, GATA3,* and *Tbet* at day 10 relative to the Th0 culture. (B) CT value from qRT-PCR gene expression analysis of the gene PU.1. Each donor is represented by a specific symbol and connected with a line. Data are from four independent experiments, each with two donors. *p<0.05, **p<0.01, ***p<0.001.

Supplementing the cultures with TGF-β induced increased production of IL-9 in the Th1 and the Th2 cultures (Fig. 1A). Additionally, FACS analysis showed an increase in the number of cells positive for IL-9, in the Th1 cultures with TGF-β (Fig. 2A). Furthermore did qRT-PCR data show an induction of the *IL-9* gene for both the Th1 and Th2 cultures with TGF-β (Fig. 3A). Of notion most IL-9 positive cells in the Th1 cultures, at day 10, co-produced IFN-γ (Fig. S3). Neutralization of TGF-β by adding anti-TGF-β to the Th1 and Th2 cultures resulted in similar level of IL-9 expression, production, and secretion compared to the classical Th1 and Th2 conditions (Figs. 1A and 3A).

To investigate if the TGF-β induced induction of IL-9 affects the expression of the Th1, Th2, and Th9 lineage specific master transcription factors T-bet, GATA-3, and PU.1 respectively, the cells were analyzed by FACS (Fig. 2B, E and F) and qRT-PCR (Fig. 3, C and D). No cultures expressed the *PU.1* gene (Fig. 3B) nor any CD4+ cells were found positive, less than 2%, for PU.1 in all FACS analysed cultures (Fig. 2B) and (data not shown). As expected for the Th2 cultures, the percentage of GATA-3-positive cells did not differ between the groups with and without TGF-β or anti-TGF-β (Fig. 2E); whereas a significant reduction in the percentage of the master Th1 transcription factor T-bet was observed in the T-bet-positive cells compared to classical Th1 culture conditions after addition of TGF-β (Fig. 2F). The *Tbet* gene expression at day 10 was unchanged by the addition of TGF-β to the Th1 cultures (Fig. 3D). The qRT-PCR *GATA3* gene results correlated with the intracellular staining, as addition of TGF-β to the classic Th2 cultures had no influence on the *GATA3 gene* expression (Fig. 3C).

The secretion of the classical Th1 and Th2 cytokines IFN-γ and IL-5 was slightly reduced when TGF-β was added to the cultures (Fig. 1, B and C). The same trend was observed in Fig. 2C and D, where the percentage of IL-13^+^ and IFN-γ^+^ cells was reduced after addition of TGF-β. Classical Th1 and Th2 conditions with and without anti-TGF-β showed comparable percentages of IFN-γ and IL-13^+^ cells (Fig. 2, C and D).

### Induction of the IL-33 receptor ST2 by IL-4 and IL-33

IL-33 has been associated with skewing of lymph-node-derived Th2 cells into a more potent subset, secreting higher levels of the Th2 associated cytokines IL-5 and IL-13 [19].

To examine if CD4+ T cells are responsive to IL-33, and further, if IL-4 and TGF-β regulate the expression of the membrane-bound IL-33 receptor (ST2), different CD4+ T cell cultures were analysed.

**Figure 4:**
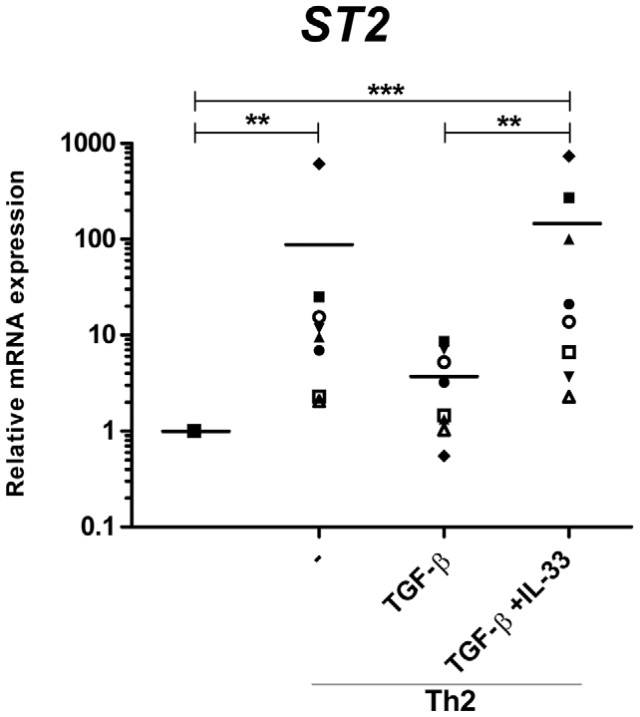
Induction of the IL-33 receptor ST2 by IL-4 and IL-33. Naïve CD4+ T cells were activated with fibroblast-bound anti-CD3/CD28 for 5 days in presence of either blocking antibodies against IFN-γ and IL-12 (Th0) or these antibodies plus IL-4 (Th2). At day 5, these cultures were restimulated and split in cultures with and without TGF-β and/or IL-33 for an additional 5 days of stimulation. qRT-PCR gene expression analysis of the relative *ST2* expression at day 10 to the Th0 culture. Each donor is represented by a specific symbol and connected with a line. Data are from four independent experiments, each with two donors. Horizontal lines represent means. **p<0.01, ***p<0.001.

Gene expression of *ST2* was induced by addition of IL-4 compared to the control Th0 culture (Fig. 4). TGF-β repressed the IL-4-induced *ST2* gene expression, whereas this effect was overcome when IL-33 was present in the culture (Fig. 4).

### IL-33 induce IL-9 secretion in Th2 cells

**Figure 5:**
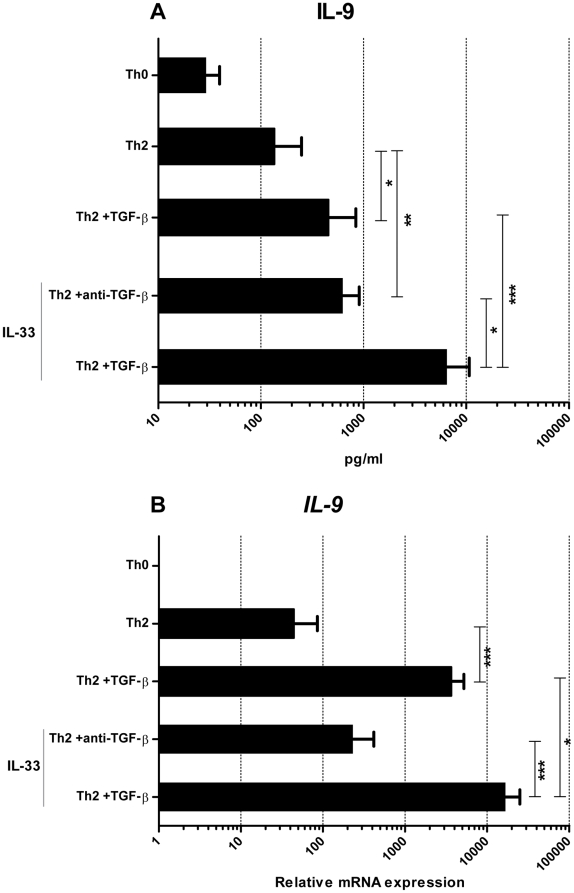
A and B. IL-33 and TGF-β induce IL-9 secretion in Th cells. Naïve CD4+ T cells were activated with fibroblast-bound anti-CD3/CD28 for five 5 days in the presence of blocking antibodies against IFN-γ and IL-12 (Th0) or these antibodies plus IL-4 (Th2). At day 5, these cultures were restimulated and split in cultures with TGF-β or anti-TGF-βplus IL-33 for an additional 5 days of stimulation. (A) Supernatant multiplex analysis of IL-9, at day 10, after restimulation with PMA and ionomycin for 6 h in the presence of Bref A for the last 4 h. (B) qRT-PCR gene expression analysis of the relative expression of *IL9* at day 10 related to the Th0 culture. Data are from four independent experiments, each with two donors. Vertical lines represent means (SEM). All tested cultures are significantly different p<0.05 or less compared to Th0 culture. *p<0.05, **p<0.01, ***p<0.001.

As already shown, TGF-β induces IL-9 in both Th1 and Th2 cultures (Figs. 1A and 3A), but surprisingly, our results (Fig. 5A) showed that IL-33 introduced to Th2 cultures causes an increase in IL-9 secretion comparable to the effect of TGF-β. This was not confirmed on the gene level (Fig. 5B). The combination of TGF-β and IL-33 potentiates the gene expression and protein secretion of IL-9 even further compared to the individual effect of the cytokines (Fig. 5A and B).

In addition to IL-33, two other IL-1 family members IL-1β and IL-18 were investigated for their capacity to induce IL-9. IL-18 further increased the TGF-β dependent IL-9 secretion (Fig. S3). IL-1β and IL-18 did however not, like IL-33 have the ability to induce IL-9 secretion in Th2 cultures with no TGF-β (Fig. S3).

As a control of endogenously produced active TGF-β, cultures with no addition of TGF-β were compared with those receiving anti-TGF-β and no differences in IL-9 gene expression, protein production and secretion were found.

### Il-33 induces IL-9 production in human basophils

**Figure 6:**
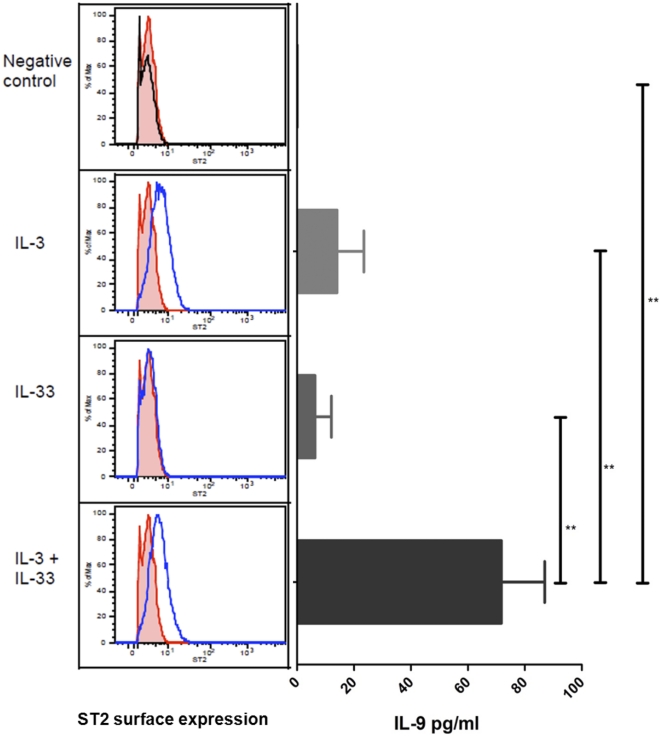
ST2 expression and IL-9 secretion from basophils stimulated with IL-3 and IL-33. Analysis of ST2 expression with FACS. Black line shows surface expression of ST2 on freshly isolated basophils at 0 h; tinted red shows negative control culture without cytokines after 24 h; blue lines show samples. Example from one donor included in both FACS and multiplex analysis is presented. In total, two independent experiments were performed, using two donors for each experiment, with similar results. Multiplex analysis of IL-9 in supernatant from a control culture or cultures with IL-3 and/or IL-33. Data are from two independent experiments, each with two donors. Vertical lines represent means (SEM). **p<0.01.

Human basophils have recently been reported to express the IL-33 receptor ST2 [6]. In agreement with previous findings, our results showed increased surface expression of ST2 on basophils stimulated with IL-3 for 24 h (Fig. 6), whereas IL-33 stimulation did not influence surface expression of its receptor (Fig. 6). Nevertheless, basophils stimulated with IL-33 released IL-9 at a level comparable to that found upon stimulation with IL-3. Moreover, there was a marked synergy between IL-3 and IL-33 on the IL-9 release (Fig. 6).

## Discussion

The notion of interleukin 9 as a Th2-type cytokine has recently been challenged by identification of the so-called IL-9-producing Th9 cells that express very few, if any, of the Th2-type cytokines (reviewed in [20]). Other sources of IL-9 may be Th17 cells and mast cells, and this suggests that the cytokine plays an important role in moderating local inflammation. A recent paper suggests that while IL-9 plays only a marginal role in modulating the inflammation in an acute asthma model, there seems to be a more profound effect in a model of chronic asthma with repeated allergen challenges. In the chronic model, IL-9 controls the remodeling process with mucus production, increased number of mast cells, increased collagen deposition, and ultimately smooth muscle cell hypertrophy [10], These and other findings may indicate that tissue-specific factors are responsible for the transition from an acute to a chronic form of inflammation, and this prompted us to study the regulation of IL-9 by tissue-specific factors such as TGF-β, IL-1β, IL-18 and IL-33.

To model lymph node T-cell differentiation, temporally separated from tissue factors, we used a 10- day culture protocol by which classical T helper-cell factors defining Th1 and Th2 were added at day 0 and the tissue-specific factors TGF-β, IL-1β, IL-18, and IL-33 after a 5-day interval. Read-out was made at day 10.

Using this human *in vitro* system, we have confirmed (Fig. 5, A and B) the results obtained in mice: IL-33 can induce IL-9 expression and secretion in T cells [21]. While others have reported [16], [17] that TGF-β is a critical factor for IL-9 induction, our results, to the best of our knowledge, for the first time indicate that TGF-β may be important but is not an absolute requirement for IL-9 induction, since cultures with IL-33 without TGF-β have markedly increased secretion of IL-9, suggesting an important role of IL-33, even though that the effect was not found significant on the gene level (Fig. 5, A). It has recently been reported [22] that IL-9 production peaks early after activation, which might explain why we find a significant increase in the supernatant IL-9 levels but no difference on its gene expression of the chosen time point. In future experiments of IL-9 regulation a kinetic study will be favorable. The TGF-β independent capacity of IL-33 to induce IL-9, do not seems to be a feature of cytokines in the IL-1 family, even though we find that IL-18 have an additive effect on IL-9 secretion (Fig. S4) and others have observed the same additive effect of IL-1β [17].

It has been reported [23], [24] that ST2 is selectively upregulated and expressed on Th2 and not on Th1 or Treg cells; thus, we did not investigate the influence of IL-33 on other Th subsets. Our results indicate that TGF-β represses the IL-4 induction of ST2; however, this effect is overcome by addition of IL-33, which could be a result of activation of both the IL-4 and IL-33 pathway overruling the TGF-β repression (Fig. 4).

The surprising induction of IL-9 in differentiated Th1 cultures was not expected, since reports have indicated a moderate dose-dependent inhibition of IL-9 secretion by IFN-γ in an *in vitro* human T cell system [17]. In contrast to this we find most IL-9 positive cells co-produce IFN-γ. It has been reported that IL-12 alone or in combination with TGF-β enhances IL-9 secretion and the percentage of IL-9^+^ CD4+ T cells when added to naïve T cells day 0, but of notice is that most of the IL-9 positive cells in these cultures were single positive for either IL-9 or IFN-γ [16], [17]. The high concentration of IL-2 in Th1 cultures supplemented with TGF-β could explain the different results; however, Beriou et al. showed a non-significant reduction in the percentage of IL-9^+^ CD4+ T cells in Th1 cultures without IL-2 compared to Th1 with added IL-2, indicating that IL-2 is important though not an absolute requirement for IL-9 induction in Th1 cultures [16]. The FACS cell number and the mRNA content (data not shown) of the Th1 cultures with and without TGF-β showed similar levels, excluding that TGF-β reduced the survival of Th1 cells as the reason for the reduced expression of *Tbet*. Culture conditions with IL-2 and TGF-β have been reported to differentiate CD4+ T cells into inducible regulatory T cells, but the signature transcription factor Forkhead box P3 was unfortunately not included in our FACS staining protocol, making a classification impossible [25].

It has been proposed that TGF-β and IL-4 induce the expression of a Th9-specific transcription factor PU.1 [14]. PU.1 and GATA proteins can physically and functionally interact, suggesting that an upregulation of PU.1 might abrogate the GATA-3 function [26], [27]. The physical interaction of PU.1 and GATA-3 could explain the lower production of IL-5 and reduced percentage of IL-13- positive cells in the Th2 cultures with TGF-β (Fig. 1B and 2B). At the gene expression level day 10 after extraneous addition of TGF-β, no cultures were found to be positive for PU.1 (Fig. 3B). As the calibrator had a CT value of approximately 30 it seems that the used probes anneal to human PU.1 and thus are functional (data not shown). When compared to the isotype control, none of the cultures analyzed by FACS for protein levels of PU.1 were found positive, suggesting that PU.1 is not the master transcription factor in our culture system. However, gene and protein analysis at earlier time points is necessary to exclude the importance of PU.1. Veldhoen et al. report an essential role for the interferon regulatory factor-4 in IL-9 secretion, though it is not specifically upregulated in Th9 cells but by most CD4+ T cells subsets, making it less suitable as a specific IL-9 marker as PU.1 [15], [28].

In agreement with other findings [12], [29], [30], TGF-β was shown to mediate repression of the Th1- and Th2-associated cytokines IFN-γ or IL-4, IL-5 and IL-13 (Figs. 1 and 2). This is further substantiated by a reduced number of CD4^+^T-bet^+^ cells in the Th1 cultures (Fig. 2F).

It has been reported that supplementing TGF-β results in unaffected or reduced expression of the Th2 master transcription factor GATA-3 [16], [17]. Partly in agreement with this, and in parallel with Wong et al., we find that the *GATA3* expression is unaffected by the supplement of TGF-β to the Th2 cultures (Figs. 2E and 3C). The unexpectedly high percentage of IL-13-positive cells in the classical Th1 cultures could be a result of the addition of IL-2, which activates STAT5, and the low strength T cell receptor activation [1]. In addition to some CD4+ T cell subsets, various other cell types have also been reported to produce IL-9, including murine mast cells [31], [32]. Since mast cells and basophils share various functional features, we wanted to test whether human basophils secrete IL-9 under the influence of IL-3, a potent activator and modulator of basophil survival and differentiation, with and without IL-33 [33], [34]. We found that both cytokines induced IL-9 secretion and, in combination, the secretion was markedly increased. This is probably due to IL-3-mediated upregulation of ST2, thereby making the basophils more responsive to IL-33 (Fig. 6). It has previously been shown that IL-33 induces expression of the main Th2-associated cytokines IL-4, IL-5, and IL-13 in human basophils, and that IL-3 act synergistically, supporting our observations [35]. One interesting aspect is the IL-9 secretion mediated by IL-33 in the absence of detectable ST2 expression. However, this is similar to previous findings [6], [35], [36] that even though surface expression of ST2 cannot be detected on freshly isolated basophils, these can still be activated by IL-33. Although we were not able to detect mRNA expression of neither ST2 nor IL-9, possibly due to the low number of cells, it should be mentioned that Suzukawa et al. [36] measured upregulation of ST2 mRNA in basophils stimulated with IL-33.

Taken together, these results indicate that the expression of IL-9 reflects a more general adaptation of the extravasated cells to specific cytokines in the microenvironment, resulting in a heterogeneous pool of IL-9-expressing cells. Therefore, the newly described association of IL-9 in the process of airway remodeling in chronic asthma highlights the need for *in vivo* experiments.

## Supporting Information

Figure S1 **Established Th1 and Th2 cultures at day 5.** Naïve CD4^+^ T cells were activated with fibroblast-bound anti-CD3/CD28 under Th0, Th1, and Th2 conditions for 5 days. Before analysis all cultures were restimulated with PMA and ionomycin for 6 h in the presence of Bref A for the last 4 h. (A, B and C) FACS analysis of percentage positive LIVE+CD4+cells for, respectively IL-9, GATA-3, and T-bet. (D, E, F and G) Multiplex analysis of supernatant concentrations of IL-9, IL-5, IFN-γ, and active TGF-β1. Data are from two independent experiments, each with two donors. Horizontal lines represent means.
(TIF)

Figure S2 **Gene expression of established Th1 and Th2 cultures at day 5.** Naïve CD4^+^ T cells were activated with fibroblast-bound anti-CD3/CD28 under Th0, Th1, and Th2 conditions for 5 days. (A, B and C) qRT-PCR gene expression analysis of the relative expression of *IL-9, GATA3,* and *Tbet* relative to the control Th0 culture stimulated with IL-2. (A and B) CT value from qRT-PCR gene expression analysis of the genes PU.1 and TGF-β1. Data are from two independent experiments, each with two donors. Horizontal lines represent means.
(TIF)

Figure S3 **Th1 cells co-produce IL-9.** Naïve CD4+ T cells were activated with fibroblast-bound anti-CD3/CD28 under classical Th1 conditions for 5 days, restimulated at day 5 with addition of TGF-β for 5 more days of stimulation. A representative dot plot diagram of the Th1 cultures percentage positive IL-9 and IFN-γ cells.
(TIF)

Figure S4 **IL-1 family member IL-18 induces IL-9 secretion in Th9 cells.** Naïve CD4^+^ T cells were activated with fibroblast-bound anti-CD3/CD28 for five 5 days in the presence of blocking antibodies against IFN-γ and IL-12 plus IL-4 (Th2). At day 5, these cultures were restimulated with TGF-β or anti-TGF-β plus IL-1β, IL-18, IL-33 for an additional 5 days of stimulation. Supernatant multiplex analysis of IL-9, at day 10, after restimulation with PMA and ionomycin for 6 h in the presence of Bref A for the last 4 h. Data are from two independent experiments, each with two donors. Vertical lines represent means (SEM). p <0.05. *p<0.05, **p<0.01, ***p<0.001.
(TIF)

## References

[1] Zhu JF, Yamane H, Paul WE (2010). Differentiation of Effector CD4 T Cell Populations.. Annual Review of Immunology, Vol 28.

[2] Saenz SA, Taylor BC, Artis D (2008). Welcome to the neighborhood: epithelial cell-derived cytokines license innate and adaptive immune responses at mucosal sites.. Immunological Reviews.

[3] Liew FY, Pitman NI, McInnes IB (2010). Disease-associated functions of IL-33: the new kid in the IL-1 family.. Nature Reviews Immunology.

[4] Allakhverdi Z, Smith DE, Comeau MR, Delespesse G (2007). Cutting edge: The ST2 ligand IL-33 potently activates and drives maturation of human mast cells.. J Immunol.

[5] Angkasekwinai P, Chang SH, Thapa M, Watarai H, Dong C (2010). Regulation of IL-9 expression by IL-25 signaling.. Nature Immunology.

[6] Pecaric-Petkovic T, Didichenko SA, Kaempfer S, Spiegl N, Dahinden CA (2009). Human basophils and eosinophils are the direct target leukocytes of the novel IL-1 family member IL-33.. Blood.

[7] Schmitz J, Owyang A, Oldham E, Song YL, Murphy E (2005). IL-33, an interleukin-1-like cytokine that signals via the IL-1 receptor-related protein ST2 and induces T helper type 2-associated cytokines.. Immunity.

[8] Lamkanfi M, Dixit VM (2009). IL-33 Raises Alarm.. Immunity.

[9] Haraldsen G, Balogh J, Pollheimer J, Sponheim J, Kuchler AM (2009). Interleukin-33-cytokine of dual function or novel alarmin?. Trends in Immunology.

[10] Kearley J, Erjefalt JS, Andersson C, Benjamin E, Jones CP (2010). IL-9 Governs Allergen-induced Mast Cell Numbers in the Lung and Chronic Remodeling of the Airways.. Am J Respir Crit Care.

[11] McMillan SJ, Xanthou G, Lloyd CM (2005). Manipulation of allergen-induced airway remodeling by treatment with anti-TGF-beta antibody: Effect on the Smad signaling pathway.. Journal of Immunology.

[12] Schmitt E, Germann T, Goedert S, Hoehn P, Huels C (1994). Il-9 Production of Naive Cd4(+) T-Cells Depends on Il-2, Is Synergistically Enhanced by A Combination of Tgf-Beta and Il-4, and Is Inhibited by Ifn-Gamma.. Journal of Immunology.

[13] Li HM, Rostami A (2010). IL-9: Basic Biology, Signaling Pathways in CD4+T Cells and Implications for Autoimmunity.. Journal of Neuroimmune Pharmacology.

[14] Chang HC, Sehra S, Goswami R, Yao WG, Yu Q (2010). The transcription factor PU.1 is required for the development of IL-9-producing T cells and allergic inflammation.. Nature Immunology.

[15] Veldhoen M (2010). Interferon Regulatory Factor 4: Combinational Control of Lymphocyte Differentiation.. Immunity.

[16] Beriou G, Bradshaw EM, Lozano E, Costantino CM, Hastings WD (2010). TGF-beta Induces IL-9 Production from Human Th17 Cells.. Journal of Immunology.

[17] Wong MT, Ye JJ, Alonso MN, Landrigan A, Cheung RK (2010). Regulation of human Th9 differentiation by type I interferons and IL-21.. Immunology and Cell Biology.

[18] Slack JL, Bi WL, Livak KJ, Beaubier N, Yu M (2001). Pre-clinical validation of a novel, highly sensitive assay to detect PML-RAR alpha mRNA using real-time reverse-transcription polymerase chain reaction.. Journal of Molecular Diagnostics.

[19] Kurowska-Stolarska M, Kewin P, Murphy G, Russo RC, Stolarski B (2008). IL-33 induces antigen-specific IL-5(+) T cells and promotes allergic-induced airway inflammation independent of IL-4.. Journal of Immunology.

[20] Noelle RJ, Nowak EC (2010). Cellular sources and immune functions of interleukin-9.. Nature Reviews Immunology.

[21] Humphreys NE, Xu D, Hepworth MR, Liew FY, Grencis RK (2008). IL-33, a potent inducer of adaptive immunity to intestinal nematodes.. Journal of Immunology.

[22] Tan C, Aziz MK, Lovaas JD, Vistica BP, Shi G (2010). Antigen-specific Th9 cells exhibit uniqueness in their kinetics of cytokine production and short retention at the inflammatory site.. J Immunol.

[23] Lecart S, Lecointe N, Subramaniam A, Alkan S, Ni DH (2002). Activated, but not resting human Th2 cells, in contrast to Th1 and T regulatory cells, produce soluble ST2 and express low levels of ST2L at the cell surface.. European Journal of Immunology.

[24] Xu D, Chan WL, Leung BP, Huang FP, Wheeler R (1998). Selective expression of a stable cell surface molecule on type 2 but not type 1 helper T cells.. Journal of Experimental Medicine.

[25] Putheti P, Awasthi A, Popoola J, Gao WD, Strom TB (2010). Human CD4(+) Memory T Cells Can Become CD4(+)IL-9(+) T Cells.. Plos One.

[26] Rekhtman N, Radparvar F, Evans T, Skoultchi AI (1999). Direct interaction of hematopoietic transcription factors PU.1 and GATA-1: functional antagonism in erythroid cells.. Genes & Development.

[27] Zhang P, Behre G, Pan J, Iwama A, Wara-Aswapati N (1999). Negative cross-talk between hematopoietic regulators: GATA proteins repress PU.1.. Proceedings of the National Academy of Sciences of the United States of America.

[28] Chang HC, Zhang SM, Thieu VT, Slee RB, Bruns HA (2005). PU.1 expression delineates heterogeneity in primary Th2 cells.. Immunity.

[29] Gorelik L, Constant S, Flavell RA (2002). Mechanism of transforming growth factor beta-induced inhibition of T helper type 1 differentiation.. Journal of Experimental Medicine.

[30] Veldhoen M, Uyttenhove C, van Snick J, Helmby H, Westendorf A (2008). Transforming growth factor-beta ‘reprograms’ the differentiation of T helper 2 cells and promotes an interleukin 9-producing subset.. Nature Immunology.

[31] Hultner L, Kolsch S, Stassen M, Kaspers U, Kremer JP (2000). In activated mast cells, IL-1 up-regulates the production of several Th2-related cytokines including IL-9.. Journal of Immunology.

[32] Wiener Z, Falus A, Toth S (2004). IL-9 increases the expression of several cytokines in activated mast cells, while the IL-9-induced IL-9 production is inhibited in mast cells of histamine-free transgenic mice.. Cytokine.

[33] Haakfrendscho M, Arai N, Arai KI, Baeza ML, Finn A (1988). Human Recombinant Granulocyte-Macrophage Colony-Stimulating Factor and Interleukin-3 Cause Basophil Histamine-Release.. Journal of Allergy and Clinical Immunology.

[34] Yamaguchi M, Hirai K, Morita Y, Takaishi T, Ohta K (1992). Hematopoietic Growth-Factors Regulate the Survival of Human Basophils Invitro.. International Archives of Allergy and Immunology.

[35] Smithgall MD, Comeau MR, Yoon BRP, Kaufman D, Armitage R (2008). IL-33 amplifies both T(h)1-and T(h)2-type responses through its activity on human basophils, allergen-reactive T(h)2 cells, iNKT and NK Cells.. International Immunology.

[36] Suzukawa M, Likura M, Koketsu R, Nagase H, Tamura C (2008). An IL-1 Cytokine Member, IL-33, Induces Human Basophil Activation via Its ST2 Receptor.. Journal of Immunology.

