# Supplementary figures and images for: IL-33 Induces IL-9 Production in Human CD4+ T Cells and Basophils
Source: PLoS One. 2011 Jul 6;6(7):e21695. doi: 10.1371/journal.pone.0021695 (PMC3130774; doi:10.1371/journal.pone.0021695)

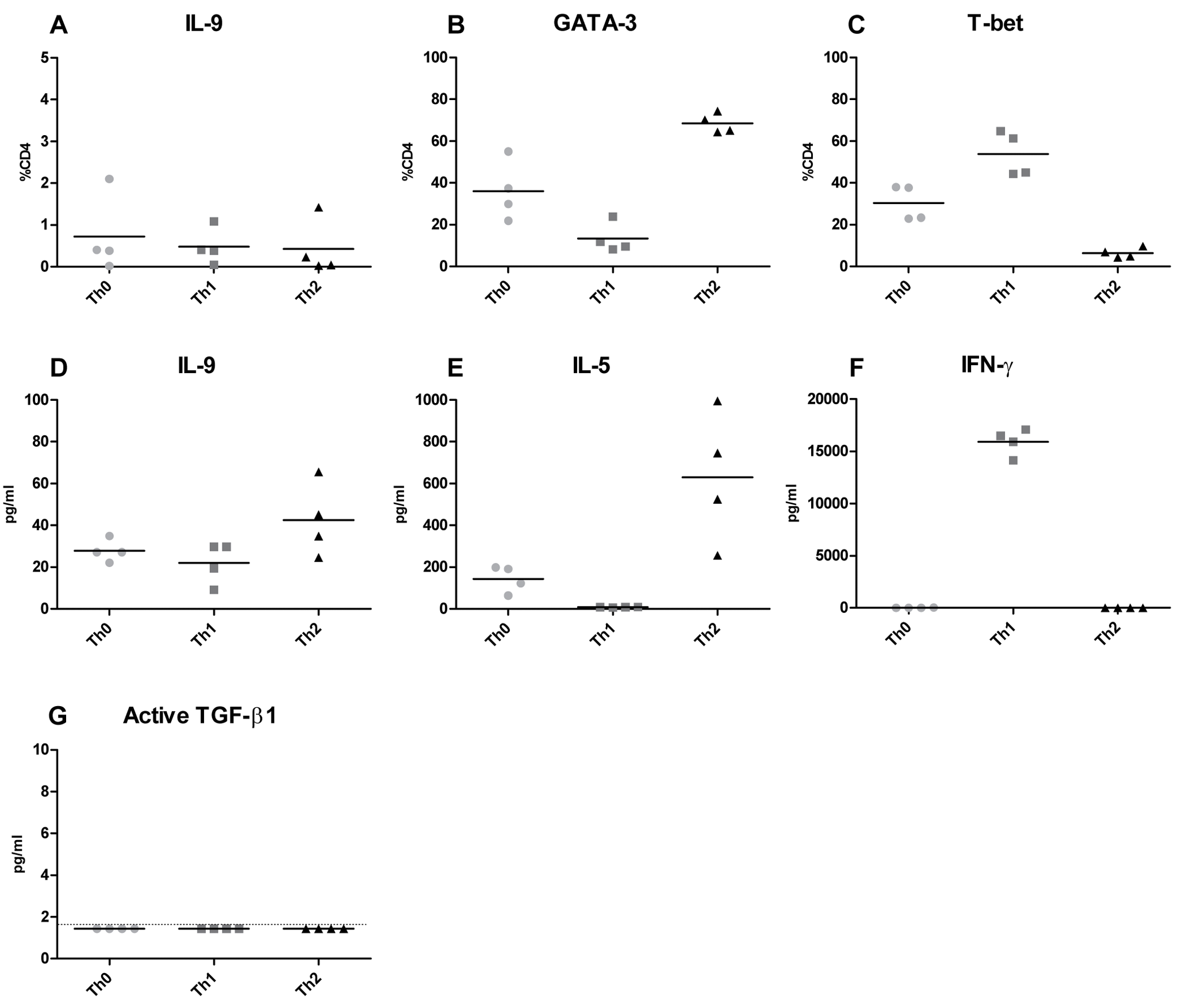

Supplement: Figure S1 — Established Th1 and Th2 cultures at day 5. Naïve CD4+ T cells were activated with fibroblast-bound anti-CD3/CD28 under Th0, Th1, and Th2 conditions for 5 days. Before analysis all cultures were restimulated with PMA and ionomycin for 6 h in the presence of Bref A for the last 4 h. (A, B and C) FACS analysis of percentage positive LIVE+CD4+cells for, respectively IL-9, GATA-3, and T-bet. (D, E, F and G) Multiplex analysis of supernatant concentrations of IL-9, IL-5, IFN-γ, and active TGF-β1. Data are from two independent experiments, each with two donors. Horizontal lines represent means. (TIF) [file pone.0021695.s001.tif]

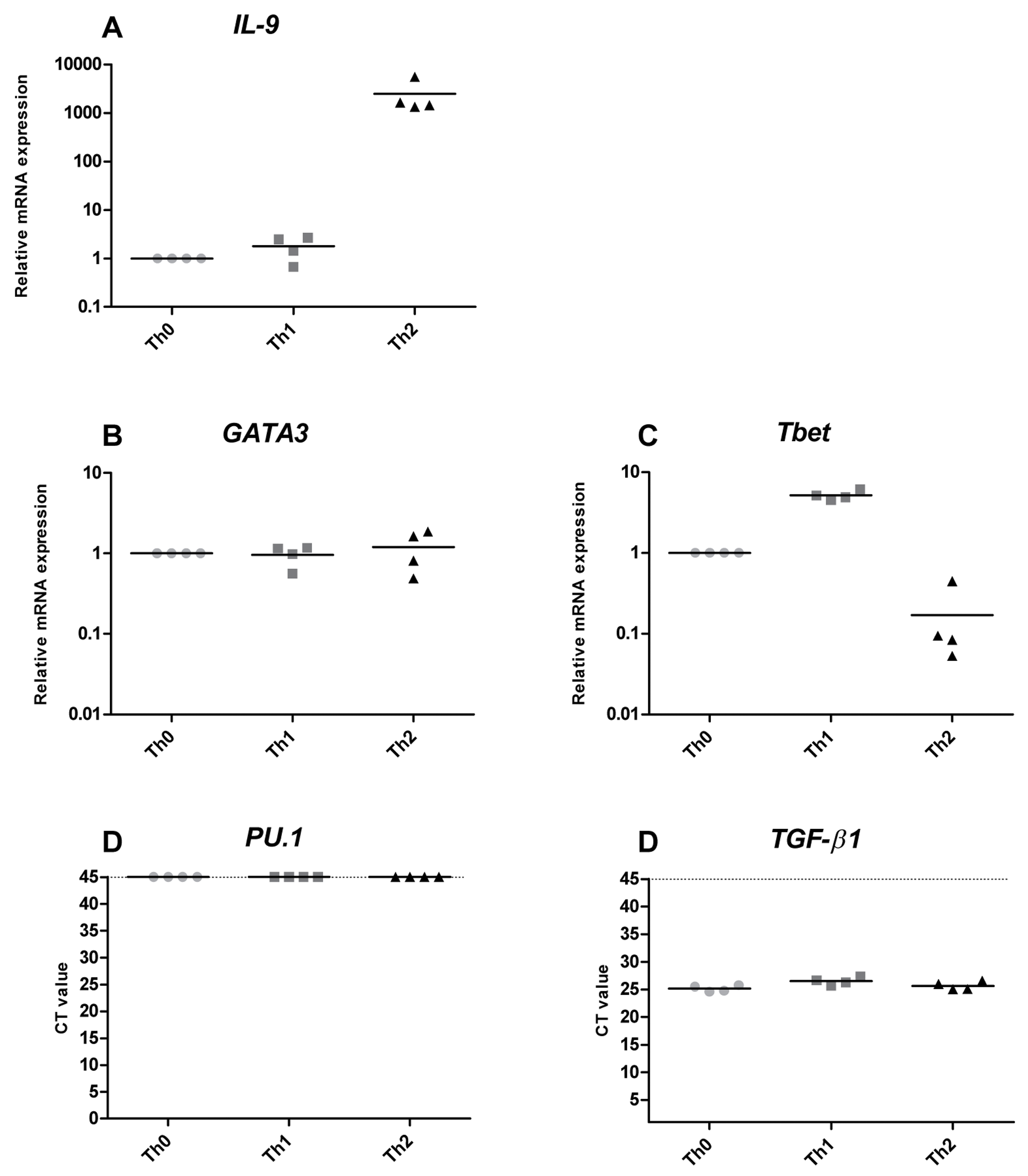

Supplement: Figure S2 — Gene expression of established Th1 and Th2 cultures at day 5. Naïve CD4+ T cells were activated with fibroblast-bound anti-CD3/CD28 under Th0, Th1, and Th2 conditions for 5 days. (A, B and C) qRT-PCR gene expression analysis of the relative expression of IL-9, GATA3, and Tbet relative to the control Th0 culture stimulated with IL-2. (A and B) CT value from qRT-PCR gene expression analysis of the genes PU.1 and TGF-β1. Data are from two independent experiments, each with two donors. Horizontal lines represent means. (TIF) [file pone.0021695.s002.tif]

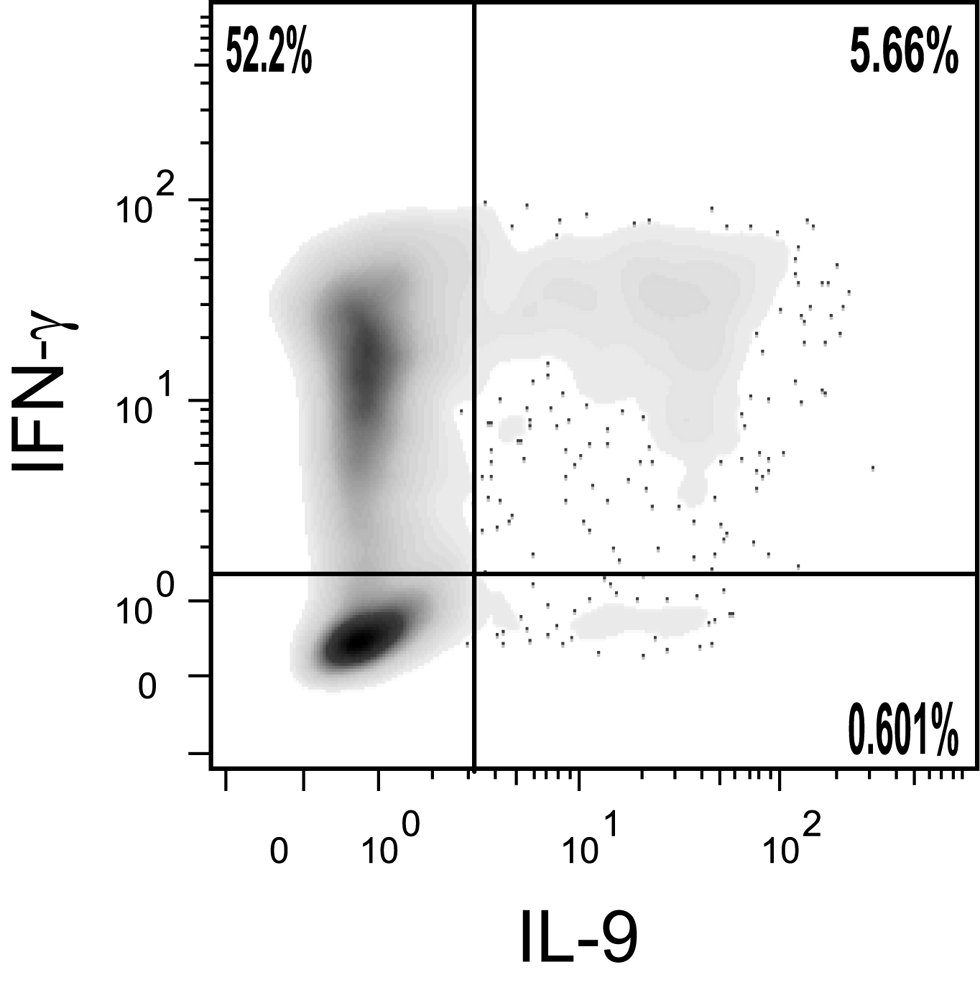

Supplement: Figure S3 — Th1 cells co-produce IL-9. Naïve CD4+ T cells were activated with fibroblast-bound anti-CD3/CD28 under classical Th1 conditions for 5 days, restimulated at day 5 with addition of TGF-β for 5 more days of stimulation. A representative dot plot diagram of the Th1 cultures percentage positive IL-9 and IFN-γ cells. (TIF) [file pone.0021695.s003.tif]

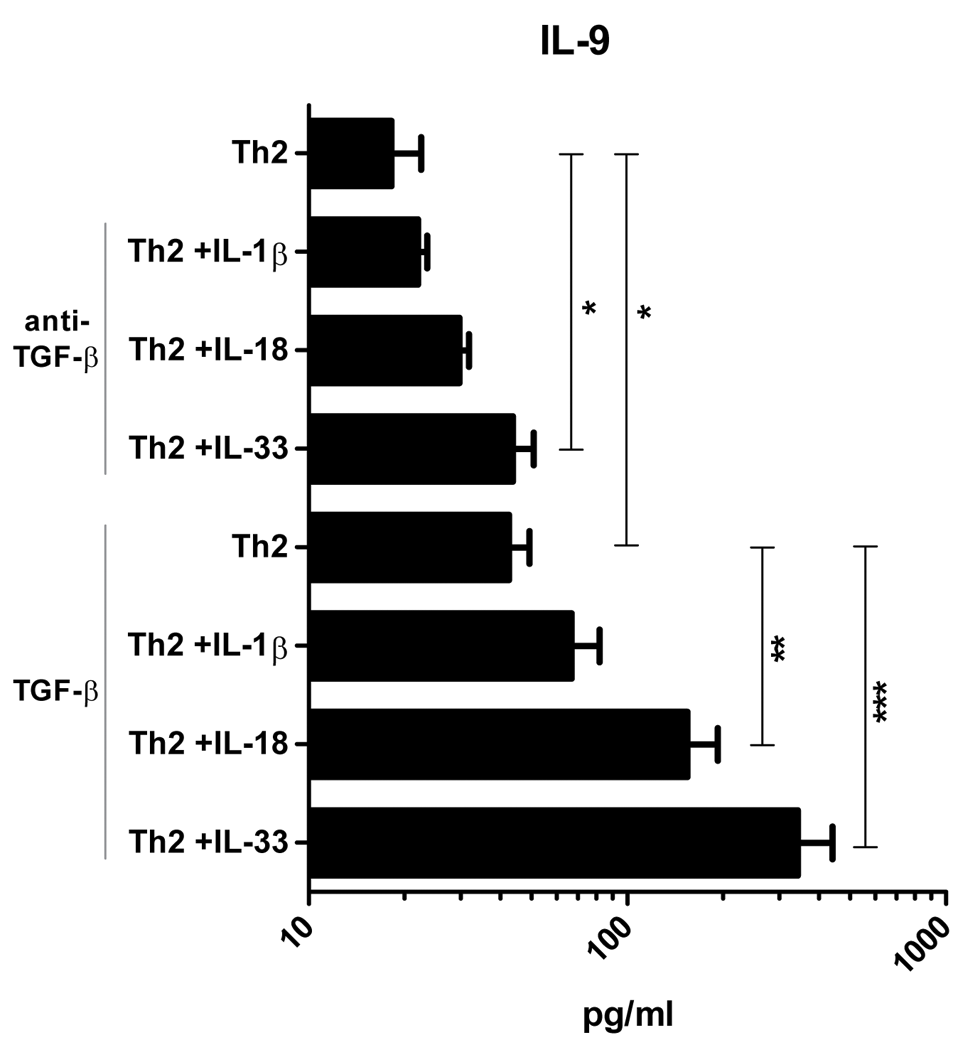

Supplement: Figure S4 — IL-1 family member IL-18 induces IL-9 secretion in Th9 cells. Naïve CD4+ T cells were activated with fibroblast-bound anti-CD3/CD28 for five 5 days in the presence of blocking antibodies against IFN-γ and IL-12 plus IL-4 (Th2). At day 5, these cultures were restimulated with TGF-β or anti-TGF-β plus IL-1β, IL-18, IL-33 for an additional 5 days of stimulation. Supernatant multiplex analysis of IL-9, at day 10, after restimulation with PMA and ionomycin for 6 h in the presence of Bref A for the last 4 h. Data are from two independent experiments, each with two donors. Vertical lines represent means (SEM). p <0.05. *p<0.05, **p<0.01, ***p<0.001. (TIF) [file pone.0021695.s004.tif]
